# Correlation between Femoral Head Lateralization and Bone Morphology in Primary Hip Osteoarthritis

**DOI:** 10.1155/2023/3158206

**Published:** 2023-08-19

**Authors:** Kenta Inagaki, Shigeo Hagiwara, Yuya Kawarai, Hiroakira Terakawa, Shuichi Miyamoto, Chiho Suzuki, Hiroyuki Yamagata, Junichi Nakamura, Seiji Ohtori, Satoshi Iida

**Affiliations:** ^1^Department of Orthopaedic Surgery, Chiba University Hospital, Chiba 260-8677, Japan; ^2^Department of Orthopaedic Surgery, Matsudo City General Hospital, Matsudo, Japan

## Abstract

**Background:**

Osteoarthritis (OA) is the most common disease of the hip in adults, and its etiology is divided into two groups: primary and secondary. Although acetabular dysplasia is the most frequent reason for total hip arthroplasty (THA) in Japan, primary OA has increased recently. Although there are two types of femoral head migration in primary OA: superior and medial, there are some patients with prominent femoral head lateralization. This study aimed at evaluating the relationship between femoral head lateralization and bone morphology of the acetabulum and proximal femur using radiographic factors in primary OA of the hip.

**Methods:**

A retrospective study was conducted between 2008 and 2017 to assess 1308 hips with OA who underwent primary THAs at our institute. The diagnostic criteria for primary OA were Crowe type 1, Sharp's angle <45°, and center-edge (CE) angle >25°. We classified patients with primary OA into two groups based on femoral head lateralization: group L with lateralization or group N without. Radiographic factors included Sharp's angle, CE angle, acetabular inclination, acetabular depth ratio (ADR), acetabular head index (AHI), and femoral neck-shaft angle (FNA), all examined on an anteroposterior pelvic radiograph. Femoral neck anteversion was calculated using computerized axial tomography.

**Results:**

Primary OA was diagnosed in 210/1308 hips (16.1%) (group L: 112 hips (8.6%); group N: 98 (7.5%)). Patient demographics were not significantly different. Radiographic factors with observed significant differences between group L and group N were the average CE angle (33.0° vs. 35.1°, respectively, *p* = 0.009), ADR (251.6 vs. 273.4, *p* < 0.001), AHI (77.2 vs. 80.4, *p* < 0.001), and FNA (136.9° vs. 134.8°, *p* = 0.012).

**Conclusions:**

This investigation suggests that primary OA with femoral head lateralization demonstrated specific identifiable radiographic characteristics in the acetabulum and proximal femur that might contribute to hip joint instability such as the dysplastic hip.

## 1. Background

Osteoarthritis (OA) is the most common disease of the hip in adults. Its etiology is commonly divided into two groups: (1) primary or idiopathic OA, in which the underlying cause cannot be defined, and (2) secondary OA in which the predisposing cause is well defined [1]. Primary OA of the hip is a frequent reason for total hip arthroplasty (THA) in Western countries [2, 3], whereas secondary OA of the hip due to acetabular dysplasia (AD) is a more typical reason for THA in Japan [4]. Several reports in the literature indicate that the prevalence of secondary OA due to AD in Asians is high [5–7], but primary OA of the hip has increased in Japan recently [4, 8]. Although primary OA is generally idiopathic, several reports reveal that femoroacetabular impingement (FAI) has been increasing and appears to be a potential precursor of idiopathic hip OA [9–11]. There is a perception that bone morphology is associated with primary OA, and several authors have reported that two types of femoral head migration can occur: (1) superior (or eccentric) and (2) medial (or concentric) [1, 12–14]. There are some patients with prominent femoral head lateralization but the correlation between femoral head lateralization and bone morphology of the acetabulum and femur has not been sufficiently evaluated in the past.

This study aimed at evaluating the relationship between femoral head lateralization and bone morphology of the acetabulum and proximal femur using radiographic factors in primary OA of the hip for the population undergoing THA.

## 2. Materials and Methods

A retrospective radiographic evaluation was performed at our institute (Matsudo City General Hospital). The research protocol for this study was approved by the Institutional Review Board of the authors' affiliated institutions in compliance with the principles of the Helsinki. Written informed consent was obtained from all participating subjects. A total of 1308 consecutive patients who underwent primary THA for hip OA from January 2008 through December 2017 were included in the study. The diagnosis of OA was determined using the criteria of Altman et al. which is hip pain, along with two of the following: (1) erythrocyte sedimentation rate (ESR) <20 mm, (2) femoral or acetabular osteophytes, and (3) joint space narrowing [15]. Two examiners (I.S. and S.C.) determined the diagnosis after discussion.

**Figure 1:**
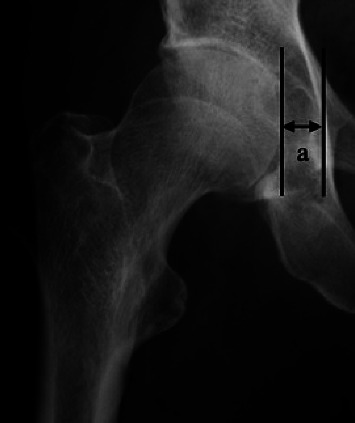
a (mm) is the distance between the ilioischial line and the medial aspect of the femoral head. When the distance was ≥10 mm, the hip was categorized as group L, and when it was <10 mm it was categorized as group N. In this case, a is 12.2 mm, so this hip is categorized as group L.

Primary OA was defined by commonly used diagnostic criteria as follows: Crowe's classification type 1 [16], Sharp's angle <45° [17], and a CE angle >25° [18]. Computed tomography (CT) was used in all cases for preoperative planning. The subjects were divided into two groups based on the lateralization of the femoral head. The lateralization group (group L) included patients in whom the distance between the ilioischial line and the medial aspect of the femoral head was ≥10 mm. In the group without lateralization (group N), the distance was <10 mm (Figure 1) [19].

**Figure 2:**
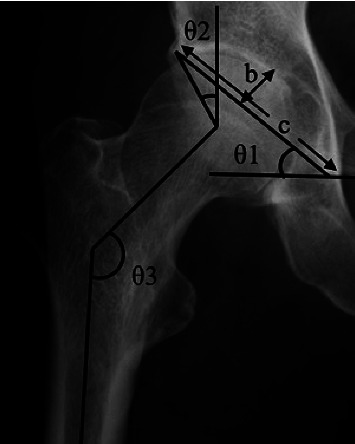
*θ*1 is Sharp's angle. *θ*2 is the CE angle. *θ*3 is the femoral shaft-neck angle (FNA). b/c is the acetabular depth ratio (ADR). Sharp's angle was the angle between the line joining the lateral aspect of the weight-bearing zone and the inferior point of the teardrop, parallel to the transverse axis of the pelvis. The CE angle was the angle between the line joining the lateral aspect of the weight-bearing zone and the center of the femoral head with the line perpendicular to the transverse axis of the pelvis. FNA was the angle between the axis of the femoral neck and femoral shaft. ADR was calculated by dividing the depth of the acetabulum by the length between the inferior teardrop point and the lateral weight-bearing zone of the center of the femoral head in the coronal plane, then multiplying by 1000.

**Figure 3:**
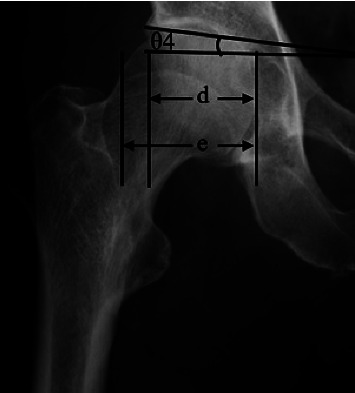
*θ*4 is the acetabular inclination (AI). d/e is the acetabulum head index (AHI). AI was the angle between the line joining the medial and lateral aspects of the weight-bearing zone and the line parallel to the transverse axis of the pelvis. AHI was calculated by dividing the distance from the medial margin of the femoral head to the lateral side of the weight-bearing zone by the width of the femoral head, then multiplying by 100.

**Figure 4:**
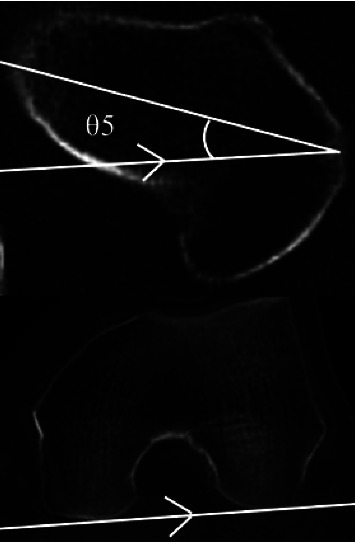
*θ*5 is the femoral neck anteversion (FNAV). FNAV was the angle between the femoral neck axis and the posterior condylar line of the distal femur.

Cases of secondary OA were excluded, and the following items were examined in cases classified as primary OA: age, sex, affected side, height, body weight, and body mass index (BMI). Sharp's angle [17], CE angle [18], acetabular inclination (AI) [20], acetabular depth ratio (ADR) [21], acetabular head index (AHI) [22], and femoral shaft-neck angle (FNA) were measured from an anteroposterior (AP) pelvic radiograph (Figures 2 and 3), and femoral neck anteversion (FNAV) was measured from an axial plane CT (Figure 4) [23, 24]. The measurement methods for each radiographic factor were as follows: Sharp's angle was the angle between the line joining the lateral aspect of the weight-bearing zone and the inferior point of the teardrop, parallel to the transverse axis of the pelvis. The CE angle was the angle between the line joining the lateral aspect of the weight-bearing zone and the center of the femoral head with the line perpendicular to the transverse axis of the pelvis. AI was the angle between the line joining the medial and lateral aspects of the weight-bearing zone and the line parallel to the transverse axis of the pelvis. ADR was calculated by dividing the depth of the acetabulum by the length between the inferior teardrop point and the lateral weight-bearing zone of the center of the femoral head in the coronal plane, then multiplying by 1000. AHI was calculated by dividing the distance from the medial margin of the femoral head to the lateral side of the weight-bearing zone by the width of the femoral head, then multiplying by 100. FNA was the angle between the axis of the femoral neck and the femoral shaft. FNAV was the angle between the femoral neck axis and the posterior condylar line of the distal femur. An AP pelvic radiograph was performed with the patient supine and their lower extremities internally rotated by approximately 15° so that the patella could be positioned in the frontal plane to maximize the length of the femoral neck. CT was performed with the patient in a supine position with the lower extremities oriented in natural rotation. Two board-certified orthopedic surgeons specializing in the hip joint evaluated all radiographic factors and used the mean value.

### 2.1. Statistical Analysis

An independent-sample Student's *t*-test and Fisher's exact test were used to compare groups L and N in terms of patient demographics and radiographic factors. We compared FNA, ADR, and FNAV between the affected and unaffected sides of unilateral OA using a paired *t*-test. The interclass correlation coefficient (ICC) of each radiographic factor and its 95% confidential interval (CI) with an absolute agreement definition were calculated. *p* < 0.05 was considered significant in all tests of statistical inference. All statistical analyses were performed using EZR (Saitama Medical Center, Jichi Medical University, Saitama, Japan), which is a graphical user interface for R (The R Foundation for Statistical Computing, Vienna, Austria).

## 3. Results

A total of 210 hips (16.1%) were diagnosed with primary OA out of 1308 hips. Among these, 112 (53.3%) hips were in group L and 98 (46.7%) in group N. There was no significant difference in demographics between the two groups (Table 1). Radiographic factors are summarized in Table 2. The average values of CE angle (33.0° vs. 35.1°, *p* = 0.009), ADR (251.6 vs. 273.4, *p* < 0.001), and AHI (77.2 vs. 80.4, *p* < 0.001) in group L were significantly lower compared to group N. On the other hand, the average value of FNA in group L was significantly larger than in group N (136.9° vs. 134.8°, *p* = 0.012). There were no significant differences in other radiographic factors including Sharp's angle, AI and FNAV. Bilateral primary OA affected 106 hips, whereas 104 hips had unilateral disease. With unilateral OA, 63 patients (60.6%) were in group L and 41 (39.4%) were in group N. The results of radiographic factors between the affected and unaffected sides in unilateral OA patients are shown in Table 3. The average ADR of the affected side was significantly less than that of the unaffected side in both groups (group L: 251.6 vs. 286.2, group N: 271.3 vs. 297.5) (*p* < 0.001). The FNA of the affected side in group L was larger than that of the unaffected side, however, it was not significantly different (*p* = 0.072). FNAV was not significantly different between the affected and unaffected sides in either group.

The ICC of each radiographic factor was as follows: Sharp angle (95% CI): 0.70 (0.55–0.84), CE angle: 0.51 (0.40–0.61), AI: 0.82 (0.69–0.96), ADR: 0.89 (0.69–0.99), AHI: 0.84 (0.75–0.94), FNA: 0.71 (0.58–0.84), and FNAV: 0.93 (0.77–0.99).

## 4. Discussion

In this study, the prevalence of primary OA of the hip was 16.1% based on the diagnostic criteria of Crowe's classification type 1 [16], Sharp's angle <45° [17], and a CE angle >25° [18]. Nakamura et al. reported in 1989 that primary OA was detected in 13 cases (0.9%) out of 2,000 consecutive cases diagnosed with hip OA [24]. The diagnostic criteria included the absence of femoral head deformity, a CE angle >19°, a Sharp's angle <45°, and an acetabular roof obliquity <15° [25]. This low prevalence could be influenced by the patient population, which was collected from a specific outpatient clinic for the treatment of acetabular dysplasia. Hoagland et al. evaluated 200 consecutive Japanese patients in Japan and 199 consecutive white American patients in the USA, all of whom were admitted for hip surgery [26]. They reported in 1985 that the prevalence of primary OA was 18% in Japanese patients and 90% in white American patients [26]. Recently, primary OA of the hip was reported to be increasing in Japan [8]. The Japanese Arthroplasty Register reported that the percentage of primary OA patients referred for primary total hip arthroplasty was 16.3% in 2013, 21.5% in 2015, and 26.6% in 2017 [8].

In 2010, Jingushi et al. conducted a multi-institutional examination of patients with hip OA who were newly admitted to the orthopedic outpatient clinic in Japan. They reported that the prevalence of primary OA was 9% (44 out of 485 hips) [4]. Thus, the prevalence of primary OA of the hip has been reported variously in Japan. The prevalence of primary OA might differ depending on the differences in diagnostic criteria and patient populations. In this study, the prevalence was 16.1% but if the hips in group L were excluded from the diagnosis of primary OA, the prevalence dropped to 7.5%, similar to the data reported by Jingushi et al. [4].

Femoral head lateralization in our study was recognized in approximately half of all primary OA cases (53.3%). Hartofilakidis and Karachalios reported that 80% (218/272) of primary hip OA patients had eccentric type (or superior migration) [1], which included superolateral or superomedial migration. Nakamura et al. reported that eight hips (62%) demonstrated superolateral types [14]. In our study, femoral head lateralization was defined as the distance between the ilioischial line and the medial aspect of the femoral head ≥10 mm. The prevalence of femoral head lateralization in primary OA in our study was similar to the result reported by Nakamura et al. [14] and less than Hartofilakidis et al., which may suggest the possibility that a racial difference might influence the prevalence of femoral head lateralization.

The etiology of femoral head lateralization in patients with primary OA is uncertain. Nakamura et al. reported that the superolateral type of primary OA develops in the subset of normal hips with a greater degree of acetabular roof obliquity [14]. Our radiographic evaluation demonstrated that the CE angle, ADR, and AHI of group L were significantly less than those of group N, and the FNA of group L was significantly larger than that of group N. Furthermore, the radiographic evaluation of the patients with unilateral primary OA demonstrated that the FNA of the affected side was slightly larger than that of the unaffected side, and the ADR of the affected side was significantly less than that of the unaffected side. The CE angle and AHI are reduced by femoral head lateralization. Regarding ADR, dysplastic hips usually have a smaller ADR than normal hips, and femoral head lateralization often occurs in dysplastic hips [27, 28]. Thus, primary OA with femoral head lateralization might be a boundary condition between primary and dysplastic OA, and the diagnostic criteria for primary OA using only the Sharp and CE angles might miss the condition. Regarding FNA, Pauwels and Maquet reported that larger femoral neck-shaft angles might induce a laterally directed joint reaction force to potentiate hip instability [27, 29]. Therefore, a larger FNA might have the potential role of femoral head lateralization in primary OA. Eventually, the smaller ADR (shallow acetabulum) and larger FNA (coxa valgus) might induce hip joint instability and correlate with the development of OA associated with femoral head lateralization. In clinical practice, cup position and stem selection might need to be modified in patients with femoral head lateralization because of their specific bone morphology.

The effect of femoral head lateralization on the clinical course in the primary OA is unclear in this cross-sectional study. In dysplastic hips, femoral head lateralization correlates strongly with the development of hip OA [1, 28]. Mimura et al. propose that femoral head lateralization induces greater hip joint pressure to maintain stabilization of the joint [28]. Hartofilakidis and Karachalios reported that hips with concentric idiopathic OA underwent THA on average 10 years after symptom onset; by contrast, eccentric hips underwent THA on average four years after symptom onset [1]. Thus, femoral head lateralization might accelerate the osteoarthritic change of the hip and advance the timing of THA. Therefore, femoral head lateralization might strongly correlate with the instability of the hip and lead to the development and progression of OA.

Our study has several limitations. First, the radiographic review was based only on AP radiographs. However, we consider that it is essential for physicians to acquire common and reliable radiographic views as well as parameters for plain radiographic assessment that can serve as a foundation for accurate diagnosis, disease classification, and surgical decision-making. Second, osteophytes of the proximal femur and acetabulum are variable, such that measurement errors in radiographic factors could occur. However, the data were reviewed by two experienced orthopedic surgeons, and measurements for cases with complex imaging findings were made following discussion. Furthermore, most of the ICCs of each radiographic factor were good. Third, the lateralization of the femoral head was determined by the distance between the ilioischial line and the medial aspect of the femoral head. The distance of 10 mm should be considered a general reference number as opposed to a strict parameter, as magnification errors and variability in patient size can influence this measurement. Fourth, FNA might be influenced by the rotation of the lower extremity [30]. However, FNA was measured using AP pelvic radiographs performed with the lower limb in internal rotation, thus placing the patella in the frontal plane. Furthermore, FNAV measured by CT in both groups was approximately 15°, and there was no significant difference between the two groups. These relatively small values of FNAV were considered to be negligible in terms of the measurement of FNA. Fifth, the stages of OA varied, and we did not distinguish among them. There is a possibility that femoral head lateralization can progress over time in accordance with the stage of OA. Sixth, the diagnostic criteria for primary OA did not include AI or AHI in this study. However, the diagnosis of primary OA was made using commonly used diagnostic criteria: Sharp's angle and CE angle.

## 5. Conclusions

Our investigation suggests that primary OA with femoral head lateralization demonstrated specific radiographic characteristics in the acetabulum (significantly smaller ADR, CE angle, and AHI) and proximal femur (significantly larger FNA), which might contribute to some hip joint instability such as the dysplastic hip.

## Figures and Tables

**Table 1:** Demographics of patients in groups L and N.

	Group L	Group N	*p*
Age (mean ± SD, y)	70.5 ± 8.4	71.2 ± 9.6	0.577
Female: male (*n*)	90 : 22	88 : 10	0.082
Right: left (*n*)	63 : 49	51 : 47	0.580
Height (mean ± SD, cm)	154.7 ± 7.4	152.7 ± 7.1	0.053
Body weight (mean ± SD, kg)	58.2 ± 9.9	56.4 ± 9.9	0.187
BMI (mean ± SD, kg/m^2^)	24.3 ± 3.7	24.1 ± 3.6	0.783

BMI, body mass index.

**Table 2:** Comparison of radiographic factors between groups L and N.

	Group L	Group N	*p*
Sharp's angle (mean ± SD, °)	38.8 ± 3.3	39.4 ± 3.7	0.192
CE angle (mean ± SD, °)	33.0 ± 5.4	35.1 ± 6.3	0.009
AI (mean ± SD, °)	4.6 ± 2.8	4.0 ± 2.6	0.177
ADR (mean ± SD)	251.6 ± 29.0	273.4 ± 30.3	<0.001
AHI (mean ± SD)	77.2 ± 5.1	80.4 ± 6.0	<0.001
FNA (mean ± SD, °)	136.9 ± 6.0	134.8 ± 5.8	0.012
FNAV (mean ± SD, °)	14.4 ± 10.7	16.5 ± 11.8	0.174

ADR, acetabular depth ratio; AHI, acetabular head index; AI, acetabular inclination; CE, center-edge; FNA, femoral neck-shaft angle; FNAV, femoral neck anteversion.

**Table 3:** Comparison of radiographic factors between affected and unaffected sides in unilateral OA.

		Affected side	Unaffected side	*p*
FNA (mean ± SD, °)	Group L	136.1 ± 6.0	134.5 ± 5.4	0.072
	Group N	135.4 ± 5.3	133.8 ± 4.4	0.101

ADR (mean ± SD)	Group L	251.5 ± 30.9	286.2 ± 31.7	<0.001
	Group N	271.3 ± 30.1	297.5 ± 34.5	<0.001

FNAV (mean ± SD, °)	Group L	15.6 ± 10.2	13.9 ± 11.4	0.206
	Group N	15.9 ± 10.8	14.7 ± 10.7	0.415

ADR, acetabular depth ratio; FNA, femoral neck-shaft angle; FNAV, femoral neck anteversion.

## Data Availability

The datasets used and/or analyzed during the current study are available from the corresponding author upon request.
